# Atrial Fibrillation in the Setting of Acute Pneumonia: Not a Secondary Arrhythmia

**DOI:** 10.31083/j.rcm2305176

**Published:** 2022-05-16

**Authors:** Anna Maisano, Marco Vitolo, Jacopo Francesco Imberti, Niccolò Bonini, Alessandro Albini, Anna Chiara Valenti, Daria Sgreccia, Marta Mantovani, Vincenzo Livio Malavasi, Giuseppe Boriani

**Affiliations:** ^1^Cardiology Division, Department of Biomedical, Metabolic and Neural Sciences, University of Modena and Reggio Emilia, Policlinico di Modena, 41124 Modena, Italy; ^2^Clinical and Experimental Medicine PhD Program, Department of Biomedical, Metabolic and Neural Sciences, University of Modena and Reggio Emilia, 41124 Modena, Italy

**Keywords:** atrial fibrillation, infection, pneumonia, CAP, critically ill

## Abstract

Atrial fibrillation (AF) is the most common arrhythmia in the setting of critically ill patients. Pneumonia, and in particular community-acquired pneumonia, is one of the most common causes of illness and hospital admission worldwide. This article aims to review the association between AF and acute diseases, with specific attention to pneumonia, from the pathophysiology to its clinical significance. Even though the relationship between pneumonia and AF has been known for years, it was once considered a transient bystander. In recent years there has been growing knowledge on the clinical significance of this arrhythmia in acute clinical settings, in which it holds a prognostic role which is not so different as compared to that of the so-called “primary” AF. AF is a distinct entity even in the setting of pneumonia, and acute critical illnesses in general, and it should therefore be managed with a guidelines-oriented approach, including prescription of anticoagulants in patients at thromboembolic risk, always considering patients’ individuality. More data on the significance of the arrhythmia in this setting will help clinicians to give patients the best possible care.

## 1. Introduction

Atrial fibrillation (AF) is a very frequent clinical condition, being the world’s most frequent arrhythmia, affecting 43.6 million people worldwide [1, 2]. Its incidence is continuously growing, with a great impact on patients’ morbidity and mortality [3, 4].

The pathophysiology of AF is multifactorial and involves numerous factors including genetic predisposition, triggers, and perpetuating elements [5]. During the years there has been a growing knowledge of the mechanisms behind the arrhythmia and their interaction. If more than 70 years ago Evans and Swann were proposing for the first time the “lone AF” term to describe a benign clinical condition without apparent risks [6], the 2020 European Society of Cardiology (ESC) guidelines on the diagnosis and management of AF suggest abandoning this type of characterization of the arrhythmia, as it can be confusing [7, 8]. As knowledge progressed, it became clear that there are underlying causes in most AF patients, and a clear precipitating factor is found in 1/3 of them [9]. Inflammation and infection are often involved in the pathogenesis of the arrhythmia, as surgery and myocardial infarction [10, 11] are as well.

Respiratory tract infections, and especially community-acquired pneumonia, are among the major causes of hospital admissions, particularly among the elderly, often correlating with adverse outcomes among frail patients [12, 13, 14, 15, 16].

Moreover, pneumonia incidence increases worldwide, boosting patients’ morbidity and mortality, with a significant increase in related healthcare costs and a rising public health problem [17, 18].

In this context, it is important to recognize that the link between pneumonia and cardiovascular diseases does not only rely on an epidemiological association (older people have an increased susceptibility to cardiovascular and pulmonary diseases), but it has many roots, often tangled one another [19, 20]. AF is the most common arrhythmia in the setting of critically ill patients affected by infectious diseases [21].

This article aims to review the association between AF and acute diseases, with specific attention to pneumonia, from pathophysiology to its clinical significance. The characteristics and main findings of seminal studies investigating the relationship between AF and the so-called “secondary precipitants”, infections and pneumonia in particular, are shown in Table 1 (Ref. [20, 22, 23, 24, 25, 26, 27, 28, 29, 30, 31, 32, 33]).

**Table 1.** **Characteristics and main findings of studies on AF in the clinical setting of critical illnesses and pneumonia**.

Study, year	Study design	Study population	Mean/Median age (years)	Follow-up	Main findings
Musher DM *et al*., 2007 [20]	Prospective study	170 patients with pneumococcal pneumonia	N/A for the entire cohort	5-year study period (2001–2005)	• Patients with pneumonia are at risk for concurrent acute cardiac events: 19.4% had ≥1 major cardiac event (12 MI; 7 AF, 1 VT; 13 HF)
					• Cardiac events increase mortality of patients with pneumonia:
					- 12.4% overall in-hospital mortality rate
					- Mortality of patients with vs. without cardiac events: 27.3% vs. 8.8%
Shaver CM *et al*., 2015 [22]	Prospective observational cohort study	1770 critically ill patients (at least 2 days in the ICU)	- AF 68 (61–77)	N/A	• AF is frequent in critical illnesses: 13% developed AF (7% new onset AF, 6% recurrent AF)
					• Factors associated with AF: male gender, caucasian race, age, cardiac disease, organ failures, disease severity, increased diastolic dysfunction, vasopressor use, greater cumulative positive fluid balance
			- No AF 56 (46–65)		• In critically ill patients AF, either new-onset or recurrent, is independently associated with increased hospital mortality (mortality of patients with AF vs. No AF: 31% vs. 17%; *p *< 0.001)
Lubitz SA *et al*., 2015 [23]	Retrospective study from the Framingham Heart Study	1409 patients with new-onset AF	74 ± 11	5.4 years	Most common AF precipitants: cardiothoracic surgery (30%), infection (23%), non-cardiothoracic surgery (20%), acute myocardial infarction (18%)
					AF recurs in most patients (including those with secondary precipitants). Recurrence rates at 5, 10 and 15 years:
					- 42%, 56% and 62% in patients with precipitants
					- 59%, 69% and 71% in patients without precipitants
					Long-term AF-related stroke (HR 1.13, 95% CI 0.82–1.57) and mortality (HR 1.00, 95% CI 0.87–1.15) risks are similar among patients with and without secondary AF precipitants
Zhu J *et al*., 2015 [24]	Retrospective study	8657 patients hospitalized in the Cardiology Department, with and without AF	- 65.8 ± 12.9 in AF group	3 years study period	AF is an independent risk factor for HAP:
					- HAP occurred in 25.64% patients with AF vs. 3.66% patients without AF
			- 60.0 ± 14.5 in No AF group		HAP is associated with increased in-hospital mortality, irrespective of AF status (6.57% HAP vs. 2.42% non-HAP)
Violi F *et al*, 2017 [25]	Prospective study	1182 patients hospitalized for CAP	73 ± 14	Up to 30 days after hospitalization	• 1/3 patients hospitalized for CAP have CVEs: HF (23.8%), AF (9.2%), MI (8%), ischemic stroke (0.9%), DVT (0.1%)
					• Factors associated with CVEs occurrence: intrahospital PSI class; age; preexisting HF
					• Intrahospital CVEs independently predict 30-day:
					- 30-days mortality: 8.7%
					- 2.4% CV deaths
Moss TJ *et al*., 2017 [26]	Retrospective cohort study	8356 patients hospitalized in the ICU	- No AF 56 (45–67)	0.8 (IQR 0.2–1.8; max 4.4) years	AF in critically ill is frequent (19%)
			- New subclinical AF 59 (46–72)		8% of all ICU admissions have new-onset subclinical/undocumented AF
			- New clinical AF 69 (61–78)		Factors associated with AF development in critically ill: age, acute respiratory failure, sepsis, postoperative state, severity of illness, haemorrhage, vasopressor requirement, valvular heart disease, gender, chronic pulmonary disease
			- Prior AF 72 (63–80)		Clinical new-onset AF is associated with increased in-hospital mortality (OR 1.63; 95% CI 1.01–2.63), but not with survival after hospital discharge
Quon MJ *et al*., 2018 [27]	Retrospective cohort study	2304 patients hospitalized for ACS, acute pulmonary disease or sepsis, with new-onset AF during admission	77.1–79.3	- 3.6 years in ACS group	• Anticoagulation’s benefit in secondary AF is less evident. Stroke rates for anticoagulant use vs no-anticoagulant use:
					- 5.7 vs. 5.3% for ACS (*p* = 0.83);
					- 4.3 vs. 3.7% for acute pulmonary disease (*p* = 0.57);
					- 7.1% vs. 5.5% for sepsis (*p* = 0.75)
				- 3.1 years in acute pulmonary disease group	• Patients with secondary AF with a prescription for anticoagulant within the first 30 days after discharge:
					- 38.4% in ACS
					- 34.1% in acute pulmonary disease
					- 27.7% in sepsis
				3.1 years in sepsis group	• The majority of patients were prescribed Warfarin. NOACs represented a minority (Dabigatran n = 32 and Rivaroxaban n = 48)
Gundlund A *et al*., 2018 [28]	Retrospective cohort study	48644 patients with infection-related and non-infection-related AF	- Infection-related AF on OAC: 77 (69–83)	5 years after hospital discharge	• Infection-related AF is associated with an increased thromboembolic risk compared to non infection-related AF: HR 1.44 (95% CI 1.16–1.78) for those initiated on OAC therapy and HR 1.17 (95% CI 1.06–1.28) for those not initiated on OAC therapy
			- Non-infection-related AF on OAC: 77 (69–83)		• OAC therapy was associated with a similar risk-reduction in AF patients with and without infection: HR for thromboembolic events was 0.75 (95% CI 0.68–0.83) for infection-related AF and 0.70 (95% CI 0.63–0.78) for non-infection-related AF
Para O *et al*., 2020 [29]	Retrospective case–control study	588 patients hospitalized in the Internal Medicine department, in SR at admission (cases: new-onset AF during hospitalization; controls: maintenance of SR)	80.02 ± 9.25	N/A	Factors independently associated with new-onset AF during hospitalization: presence of a number of comorbidities ≥3 (OR = 1.52), sepsis as a reason of hospitalization (OR = 2.16) and glycemic value at the admission ≥130 mg/dL (OR = 1.44)
Cangemi R *et al*., 2019 [30]	Prospective study	472 patients hospitalized for CAP	- CAP without AF 69.3 ± 17.2	hospitalization period	9.5% of patients hospitalized for CAP had a new episode of AF within 24 to 72 hours from admission
			- CAP with AF 79.7 ± 9.6		Independent predictors of AF occurrence in patients with CAP:
					- history of paroxysmal AF (OR 11.7; 95% CI 5.8–23.7)
					- enlarged LAAi (OR 5.4; 95% CI 2.5–11.9)
					- concentric left ventricular hypertrophy (OR 2.2; 95 CI 1.1–4.6)
					55.8% re-established sinus rhythm upon discharge
Pieralli F *et al*., 2019 [31]	Prospective study	468 patients hospitalized for CAP	75.5 ± 14.4	hospitalization period	10.3% patients had new onset AF during hospitalization
					${\mathrm{CHA}}_{2}$${\mathrm{DS}}_{2}$-VASc score is an accurate and independent predictor of new onset AF in patients with CAP:
					- ${\mathrm{CHA}}_{2}$${\mathrm{DS}}_{2}$-VASc score >3 is independently associated with new onset AF (HR 2.3; 95% CI 1.19–4.44)
Gundlund A *et al*., 2020 [32]	Retrospective registry-study	- 30307 patients with infection-related AF	79 (71–86)	1 year after hospital discharge	• 36% of patients with infection-related AF had a new hospital contact with AF during the first year after discharge
					• Infection-related AF is associated with an increased long-term risk of AF (HR 25.98, 95% CI 24.64–27.39) and thromboembolic events (HR 2.10, 95% CI 1.98–2.22) compared with infection without AF
		- 90912 patients with infection without AF			• Differences across infection types exist:
					- GI infections have the lowest odds of developing AF, but the highest risk of AF recurrence and thromboembolic events;
					- pneumonia have the highest odds of developing AF, but the lowest risk of AF recurrence and thromboembolic events
Wang *et al*., 2020 [33]	Multi-institutional longitudinal study based on electronic medical records	10723 patients with a newly diagnosed AF	67.9 ± 9.9	2.5 [IQR: 0.8, 5.4] years	19% of patients had an acute precipitant
					Most common AF precipitants: cardiac surgery, pneumonia, non-cardiothoracic surgery
					AF after acute precipitants tends to recur, but with a lower risk than patients without precipitants:
					- 41% vs. 52% 5-years recurrence rates in AF with vs. without precipitants (HR: 0.75, 95% CI: 0.69–0.81)
					Lowest recurrence risk in postoperative AF (32% in cardiac surgery, 39% in non-cardiothoracic surgery)
					AF recurrence is associated with increased stroke (HR: 1.57, 95% CI: 1.30–1.90) and mortality (HR: 2.96, 95% CI: 2.70–3.24) risk
Abbreviations: ACS, acute coronary syndrome; AF, atrial fibrillation; CAP, cap community acquired; CI, confidence interval; CV, cardiovascular; CVEs, cardiovascular events; NOAC, non-vitamin K oral anticoagulants; DVT, deep venous thrombosis; HAP, hospital-acquired pneumonia; HF, heart failure; HR, hazard ratio; GI, gastrointestinal; ICU, intensive care unit; IQR, interquartile range; LAAi, indexed left atrial area; MI, myocardial infarction; N/A, Not Available/Not Applicable; No AF, absence of AF;OR, odds ratio; PSI, Pneumonia Severity Index; VT, ventricular tachycardia.					

## 2. Atrial Fibrillation and Pneumonia: Not Just an Epidemiological Association

**Fig. 1.**
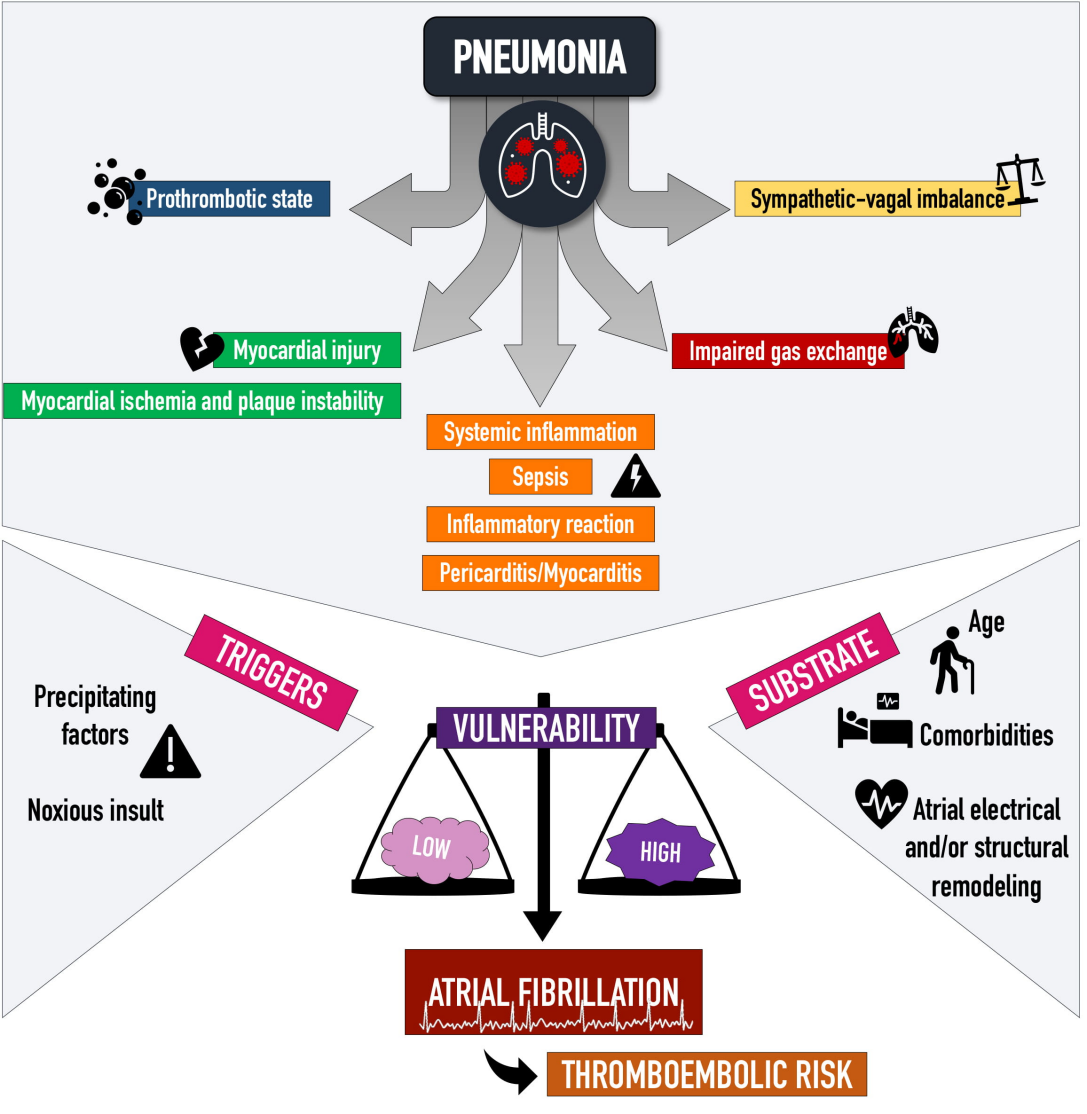
**Pathophysiology of atrial fibrillation in the setting of pneumonia**. Atrial fibrillation pathogenesis in the setting of pneumonia is multifactorial. The infection triggers inflammation and a prothrombotic state, hemodynamic changes and sympathetic-vagal imbalances, and it also acts as a noxious direct insult. These factors can interact in an already predisposed environment, thus contributing to a higher or lower probability of arrhythmia onset.

Despite medicine progresses and new therapies, a considerable proportion of patients with community-acquired pneumonia still has cardiovascular complications, with a trend that is not declining [25]. Among these, beyond acute myocardial infarction and heart failure, arrhythmias are a frequent event and AF above all [19, 25], with a pathogenesis that is multifactorial (Fig. 1).

It is clear that pneumonia and AF share many common risk factors, with older age, chronic obstructive pulmonary disease and chronic cardiac diseases being among of the most important [13, 34].

Given this epidemiological link, it is well established that pulmonary infections themselves contribute to a pro-arrhythmic environment through oxidative stress, cytokine release and changes in the hemodynamic status of the patient [35]. As known, bacterial endotoxins could promote an hyperdynamic cardiovascular status which leads to a decrease in left ventricular ejection fraction and a consequent increase in telediastolic pressures [36].

In critically ill and septic patients, a wide number of microvascular and hemodynamic dysfunctions, including microvascular thrombosis, loss of cellular integrity and ventricular fluid overload, are responsible for elevations of cardiac troponin and natriuretic peptides, which, overall, can cause myocardial dysfunction and have a proarrhythmic effect [21, 37].

From a hemodynamic point of view, sepsis-related tachycardia and anemia can contribute to myocardial ischemia and widen the gap between oxygen demand and offer, which is especially significant in pulmonary infections. In addition, hypoxia triggers pulmonary vasoconstriction and pulmonary arterial pressure elevation, resulting in increased right ventricular afterload [37].

Of note, a correlation between pulmonary infections and myocarditis has been found in up to 38% of the population with pneumonia in a small study by Saphir *et al*. [38], and sometimes antibiotic therapies, such as sulphonamides, have increased this pathological association. Interestingly, no relationship between the severity of pneumonia and myocardial changes was found.

Paraphrasing a mainstay theory in AF [39], it could be affirmed that inflammation begets AF, and it does so through an atrial structural and electrical remodelling that hesitates in the so-called atrial cardiomyopathy [40, 41, 42].

From another perspective, Zhu *et al*. [24] conducted a case-control study to assess if AF may be a risk factor for pneumonia occurrence. In their study, AF turned out to be an independent risk factor for hospital-acquired pneumonia even after adjustments for multiple variables, including age. The authors [24] suggest that this observation could be related to arrhythmia-induced hemodynamic changes, as such reduced cardiac output caused by the irregular rhythm and pulmonary congestion, which could make patients more prone to pulmonary infections. This statement would be supported also by the fact that paroxysmal AF had a more significant association with hospital-acquired pneumonia than non-paroxysmal AF: lesser the time of arrhythmia onset (paroxysmal vs. not), lesser the time for adapting to arrhythmia hemodynamic changes, leading to consequent worst clinical conditions and more infective predisposition in paroxysmal AF patients [24].

## 3. Prognostic Significance of Atrial Fibrillation in the Setting of Pneumonia

As reported by Violi *et al*. [25], patients with pneumonia who develop cardiovascular complications have more comorbidities (such as hypertension, diabetes mellitus, dyslipidemia, peripheral artery disease, stroke and chronic kidney disease) and a more severe pulmonary infection. In their multicentre study on 1182 patients hospitalized for community-acquired pneumonia, one-third developed cardiovascular events and this association had a negative impact on prognosis, with a 5-fold increase in 30-day community-acquired pneumonia-related mortality [25]. These data are consistent with similar studies on critically ill patients showing that new-onset AF is common and associated with longer hospital stay and higher mortality, both in-hospital and after discharge [22, 26, 43].

Nevertheless, patients with AF are increasingly older and with more comorbidities, leading to an overall increased thromboembolic risk compared to the past decade. Even if anticoagulant therapy was correctly employed and thromboembolic events decreased, the all-cause and cardiovascular mortality have significantly increased [44, 45].

An important finding of the last years, derived from an analysis of the Framingham cohort [23] and confirmed by other studies [46, 47, 48, 49, 50, 51]: patients with new-onset AF in the setting of secondary precipitants (i.e., infection, surgery, acute myocardial infarction) are at risk of arrhythmia recurrence. When compared to patients who develop AF without precipitants, those with “secondary” AF had a lower risk of recurrence (62% in AF with precipitants vs. 71% in AF without precipitants) but similar stroke and mortality risks. These findings underscore that the prognostic significance of these two “categories” of AF is similar, and they should therefore be considered as two branches of the same tree [23, 33].

## 4. Predicting New-Onset Atrial Fibrillation in Critically Ill Patients

Clarified the relationship between pneumonia and AF, several attempts have been made to search for factors that could predispose to AF development in this setting. On a larger perspective, efforts were directed to identify patients in whom a more accurate electrocardiographic monitoring should be performed (particularly because the vast majority of patients with infections are hospitalized in medical wards other than cardiology, and continuous ECG monitoring is not available for all).

From a clinical point of view, it has been noticed that risk factors for AF development in critically ill patients are similar to those of patients without a clear AF precipitant: the higher the number of comorbidities, the higher the probability of developing AF. At the same time, the weight of single diseases in the pathogenesis of the arrhythmia is low, while a greater role is played by the acute illness, as suggested by the lower proportion of structural heart abnormalities in critically ill patients [29].

Among those who do have cardiac morphological alterations, concentric left ventricular hypertrophy and enlarged left atrium indexed area have been found to be independently associated with an increased risk of AF in patients with community-acquired pneumonia as compared to the general population [30, 52].

In this perspective, Pieralli *et al*. [31] in 2019 investigated the role of the ${\mathrm{CHA}}_{2}$${\mathrm{DS}}_{2}$-VASc score in predicting incident AF. In a population of patients hospitalized for community-acquired pneumonia with no previous documentation of AF, the ${\mathrm{CHA}}_{2}$${\mathrm{DS}}_{2}$-VASc score was able to predict new-onset AF, both as a per se parameter, and especially if the score was >3. In this study, at the univariate analysis the CURB-65 (Confusion, Urea, Respiratory rate, Blood pressure, age >65 years) parameter could predict new-onset AF, although it was not confirmed at the multivariate analysis. These observations support the idea that a major role in the onset of the arrhythmia is played by AF risk factors, and that the acute illness acts as a promoting element in an already predisposed pabulum: in fact, only the ${\mathrm{CHA}}_{2}$${\mathrm{DS}}_{2}$-VASc score (which is a score that summarizes AF common risk factors) was able to predict arrhythmia development, while CURB-65 (that focuses on the evaluation of pneumonia severity) was not.

## 5. Atrial Fibrillation Calls for Anticoagulation. Is This Still True for Atrial Fibrillation during Infection? 

Literature regarding anticoagulation therapy in new-onset AF during infections is sparse.

Addressing this question, the thromboembolic risk associated with AF in this setting has to be evaluated, as the risk of arrhythmia recurrence and its prognosis. As previously mentioned, more than 30% of patients with AF during infection experienced arrhythmia recurrence during the first year [32]. Moreover, the thromboembolic risk of patients with infection who developed AF was double as compared to patients who went through the infection free from the arrhythmia [32].

Concerning the risk of new-onset AF, not all infections are the same. For example, pulmonary infections have the highest risk of new-onset AF, with an odds ratio of 3.27 as compared to gastro-intestinal (GI) tract infections (with the lowest risk) [32]. Of note, these two sites of infection have the opposite relationship regarding thromboembolic risk, which is highest in GI tract infections and lowest in pulmonary ones. The different elements involved and their proportional contribution in AF genesis well explain this observation: in patients with multiple AF risk factors, an isolated infection is sufficient to trigger the arrhythmia, and vice versa, pulmonary infections (which have a major impact on hemodynamic and heart function) can elicit AF even in less predisposed patients.

Interestingly, initial registration trials for direct oral anticoagulant drugs and warfarin excluded patients in which AF was considered due to a reversible disorder, as well as infective diseases [53, 54, 55, 56, 57].

To these days, few studies have directly evaluated anticoagulant therapy in the particular setting of acute infections. A remarkable one is from Gundlund *et al*. [28], in which the anticoagulant treatment in the new-onset AF population reduced thromboembolic risk in the infection-related cohort as the same as in the non-infection-related.

On the other hand, Quon *et al*. [27] in 2017 published a retrospective analysis of a cohort of patients with so-called secondary AF, developed during hospitalization for acute coronary syndromes, pulmonary diseases (including pneumonia) and sepsis, and they didn’t find a benefit from anticoagulation in terms of thromboembolic risk, while the bleeding risk was increased. As the authors pointed out, these results may partly be explained by the difference in stroke and bleeding risk of this secondary AF population as compared to the risk in primary AF. In addition, the vast majority of anticoagulated patients was on warfarin. These presented discordant data suggest that the field of anticoagulation therapy for new-onset AF during infection and acute illnesses needs to be better explored.

Most of literature evidence concerning anticoagulant strategies for new-onset AF during infections come from old cohorts. Although many changes in AF classification have taken place in the last different AF guidelines, the primary or secondary AF diagnosis had a profound impact on AF management in the past. This classification led to the general clinicians’ attitude to be less prone in the anticoagulation prescription for new-onset AF patients, especially in the setting of a significant transient promoting factor, as infectious diseases are. In the study by Quon *et al*. [27], which included patients from 1998 to 2015, almost one-third of patients initiated anticoagulant therapy. In a retrospective analysis performed by Arunachalam *et al*. [58] among patients with sepsis and septic shock, with 23% being lung infections, 44% of patients with new-onset AF during sepsis was discharged without anticoagulant therapy.

The 2019 European Heart Rhythm Association (EHRA) consensus tried to standardize the management of critically ill and post-surgery patients’ arrhythmias [21]. This paper underscores the complex and close link between arrhythmias, particularly the supraventricular ones, and sepsis/pulmonary infections. Furthermore, it emphasized the importance of promptly recognize and treat the primary cause of acute illness to contribute, in some cases, to arrhythmia self-termination. From a therapeutic perspective, when AF episodes occur, as the same for “primary AF”, thromboembolic and bleeding risk should be assessed, and subsequent anticoagulant therapy initiated as indicated in ESC guidelines [7]. Moreover, in the medications decision, it is necessary to consider the patient frailty profile and try the best to remove modifiable bleeding risk factors [5, 7, 8, 59].

**Fig. 2.**
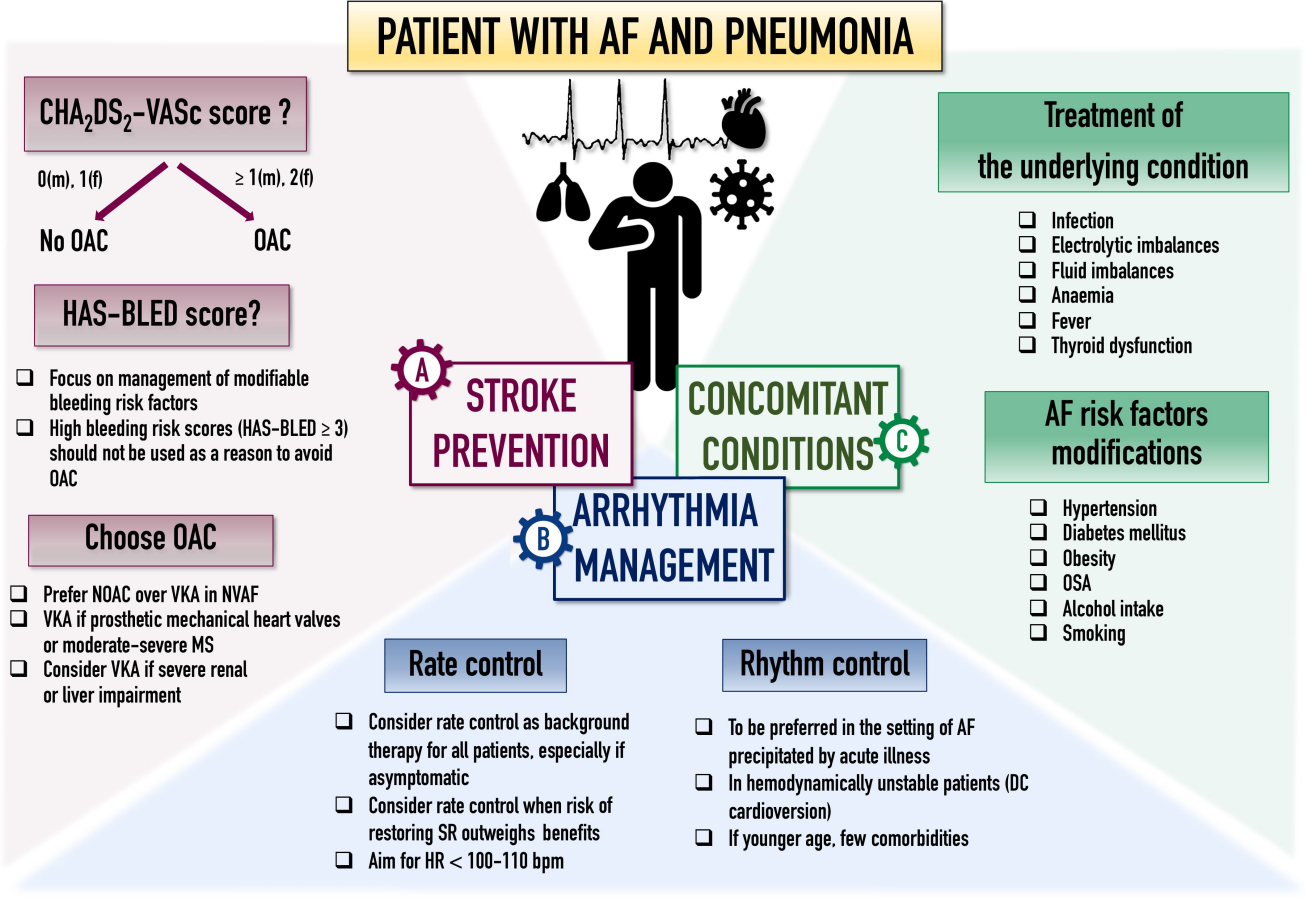
**Suggested management in patients with AF in the setting of pneumonia according to the ABC pathway**. Abbreviations: ABC, Atrial fibrillation Better Care (ABC) pathway; AF, atrial fibrillation; DC, direct cardioversion; HR, heart rate; MS, mitral stenosis; NOAC, non-vitamin K oral anticoagulants; OAC, oral anticoagulants; OSA, obstructive sleep apnoea; SR, sinus rhythm; VKA, vitamin K antagonist.

This approach is similar to that recommended for postoperative AF, which is not considered a transient and benign entity anymore, and it has now been demonstrated to be correlated with higher stroke and mortality risk [7, 11]. Indeed, in the specific setting of acute pneumonia observational data indicate that new-onset AF is associated with AF recurrences, as well as a risk of stroke and mortality. Even if no randomized controlled studies have investigated this specific setting, the consensus document from EHRA recommends to follow the general approach to thromboprophylaxis for acute illness, i.e., to assess stroke/thromboembolic risk, taking into account that low risk patients (${\mathrm{CHA}}_{2}$${\mathrm{DS}}_{2}$-VASc 0 in male and 1 in females) do not need long-term anticoagulation [8]. An advisable approach, as suggested by ESC guidelines [7] for AF occurring after non cardiac surgery is that long-term treatment with oral anticoagulants should be considered taking into account the anticipated net clinical benefit of anticoagulation and patient preferences [7]. These data highlight that AF should be managed with a holistic and integrated approach [60] (Fig. 2). Beside anticoagulation and rate/rhythm control, management of comorbidities has a significant impact on patients’ prognosis and a comprehensive approach to AF characterization (using the novel 4S-AF scheme) and treatment (following the ‘Atrial fibrillation Better Care’ pathway) has proven benefits [61, 62, 63].

Finally, when considering arrhythmia duration in the decision making process, it should be kept in mind that clinical atrial fibrillation diagnosis, as defined by 2020 ESC guidelines [7], requires a standard 12-lead ECG recording or a single-lead tracing of >30 seconds with a heart rhythm showing irregularly irregular R-R intervals, absence of distinct repeating P waves, and irregular atrial activation. Noteworthy, guidelines suggest treating AF irrespectively of its type (i.e., paroxysmal versus non-paroxysmal), particularly when deciding on anticoagulation. Stroke risk should be evaluated based on patient’s risk profile (expressed by the ${\mathrm{CHA}}_{2}$${\mathrm{DS}}_{2}$-VASc risk score) and not on AF type or burden. The relationship between AF and stroke risk is more complex than just a direct association and it does not increase linearly from the paroxysmal to the permanent AF pattern. From a wider perspective, even if the evidence is not completely concordant, in clinical practice there is no difference in thromboembolic risk between paroxysmal and non-paroxysmal AF [64, 65, 66]. This notion remains true even in the context of infection-related AF. On the other hand, AF burden (i.e., the duration of AF episodes) needs to be considered in the management of subclinical AF, defined as AF episodes detected by insertable cardiac monitors or wearable devices. In this context, ESC guidelines [7] suggest considering device-detected arrhythmia burden, combined with classical stroke risk scores, since longer episodes are associated with higher stroke risk and adverse events in general [67, 68, 69, 70]. Management of subclinical atrial fibrillation in the setting of an infection is still a matter of investigation and is a topic beyond the purpose of this review.

## 6. COVID-19 and Atrial Fibrillation

Since 2020, SARS-CoV-2 infection has developed as a pandemic disease, affecting millions of people worldwide, profoundly impacting morbidity and mortality [71, 72, 73, 74]. As a result, a considerable amount of literature has been produced about the pathophysiology of COVID-19 disease and its impact on multiple organs, including the cardiovascular system [75]. Table 2 (Ref. [76, 77, 78, 79, 80, 81, 82]) summarizes the main characteristics of the most recent studies that analyzed the effects of COVID-19 on cardiovascular events and AF.

**Table 2.** **Characteristics and main findings of studies on COVID-19 and cardiovascular events and AF**.

Study, year	Study design	Study population	Mean/Median age (years)	Follow-up	Main findings
Bhatla A *et al*., 2020 [76]	Retrospective, single center	700 COVID-19 hospitalized patients	50 ± 18	74-day period	• Incidence of cardiac events in COVID-19 patients is not only the consequence of the infection, but it is mainly dependent on the severity of the disease
					• In-hospital mortality: 4.3%
					- AF was associated with in-hospital mortality (OR 6.73; 95% CI 2.52–17.98)
					• Factors associated with arrhythmias:
					- Admission to the ICU (OR for AF 4.68; 95% CI 1.66–13.18; OR for NSVT 8.92; 95% CI 1.73–46.06)
					- Age (OR for AF 1.05; 95% CI 1.02–1.09)
					- Heart failure (OR for bradyarrhythmias 9.75; 95% CI 1.95–48.65)
Sala S *et al*., 2020 [77]	Prospective, single center	132 stable COVID-19 hospitalized patients	65 ± 14	Single-day snapshot	• Low prevalence of arrhythmias among clinically stable COVID-19 patients
					• 9% had arrhythmic events (12 patients): 8/12 AF; 4/12 supraventricular tachyarrhythmias
					• No differences between swab + patients and those with CT scan-proven pneumonia or requiring CPAP for a more severe illness
					• Factors associated with AF development: older age; at least one pre-existing risk factor
Rav-Acha M *et al*., 2021 [78]	Retrospective, single center	390 COVID-19 hospitalized patients	57.5 (43–74.3)	6 (2–10.25) days of hospitalization	• 7.2% (28 patients) had arrhythmias during hospitalization
					• The most frequent arrhythmia amongst COVID-19 patients is AF (20/28)
					• Factors associated with new tachyarrhythmias:
					- Age (OR 1.04, 95% CI 1.01–1.08)
					- CHF (OR 4.78, 95% CI 1.31–17.48)
					- Syncope/Palpitation (OR 7.57, 95% CI 1.27–45.17)
					- Disease severity (OR 8.91, 95% CI 1.68–47.29 for critical illness)
Romiti GF *et al*., 2021 [79]	Metanalysis of studies reporting AF prevalence in COVID-19 patients	31 studies	N/A	N/A	• Prevalence of AF in COVID-19 patients: 8.0% of patients had AF
					• Factors associated with AF: age; male gender; hypertension; DM; CAD; CHF; critical COVID-19 disease
		187,716 COVID-19 hospitalized patients			
					• AF is associated with
					- increased all-cause mortality risk (OR 3.97, 95% CI 2.76–5.71)
					- in-hospital mortality (OR 3.52, 95% CI 2.44–5.10)
					- 30-days mortality (OR 7.34, 95% CI 3.11–17.34)
Lip GYH *et al*., 2021 [80]	Prospective observational	280,592	72.5 (SD 9.9)	8-month study	• COVID-19 status has a stronger association with incident AF than classic cardiovascular risk factors
				period	
		- with and without incident COVID-19 infection			
		- with cardiovascular and non-cardiovascular multimorbidities			• Incidence of AF in the new COVID-19 cases was 2.5% vs. 0.6% in the non-COVID-19 cases
		- without AF history			• Factors associated with incident AF:
					- COVID-19 infection (OR 3.12; 95% CI 2.61–3.710);
					- congestive HF (OR 1.72; 95% CI 1.50–1.96);
					- CAD (OR 1.43; 95% CI 1.27–1.60);
					- VHD (OR 1.42; 95% CI 1.26–1.60)
Rivera-Caravaca JM *et al*., 2021 [81]	Retrospective observational	1270 outpatient with COVID-19 and cardiometabolic disease	67.7 ± 12.8	Up to 30-days after COVID-19 diagnosis	• In COVID-19 outpatients with cardiometabolic diseases, prior use of NOAC therapy vs. VKA therapy was associated with a lower risk of thrombotic outcomes (both arterial and venous), without increasing bleeding risk:
		- 635 on VKAs			- higher risk of ischemic stroke/TIA/SE at 30-days after COVID-19 diagnosis in VKA users vs. NOAC users (HR 2.42, 95% CI 1.20–4.88);
		- 635 on NOACs			- similar risk between VKA and NOACs patients for all-cause mortality, ICU admission/MV necessity, ICH/gastrointestinal bleeding
Denegri A *et al*., 2021 [82]	Retrospective, single center	201 COVID-19 hospitalized patients	68.5 ± 14.7	30-days	• Higher survival in COVID-19 pneumonia patients in sinus rhythm at hospital admission
					• 20.9% 30-day mortality
					• ECG at admission predictors of increased mortality:
					- AF (OR 12.74, 95% CI 3.65–44.48)
					- ST segment depression (OR 5.30, 95% CI 1.50–18.81)
					- QTc-interval prolongation (OR 3.17, 95% CI 1.24–8.10)
					• Independent predictors of increased survival:
					- sinus rhythm (HR 2.7, 95% CI 1.1–7.0)
					- LMWH (HR 8.5, 95% CI 2.0–36.6)

Abbreviations: AF, atrial fibrillation; OR, odds ratio; CAD, coronary artery disease; CHF, congestive heart failure; CI, confidence interval; CPAP, continuous positive airway pressure; CT, computed tomography; DM, diabetes mellitus; NOACs, Non-vitamin K oral anticoagulants; HF, heart failure; HR, hazard ratio; ICH, intracranial haemorrhage; ICU, intensive care unit; LMWH, low molecular weight heparin; MV, mechanical ventilation; OR, odds ratio; QTc, corrected QT interval; SD, standard deviation; SE, systemic embolism; TIA, transient ischemic attack; VHD, valvular heart disease; VKAs, vitamin K antagonists.

At the beginning of this pandemic, Libby and Lüscher [83] published an interesting review in which they referred to COVID-19 as an “endothelial disease” strictly connected to inflammation. Cardiovascular involvements and cardiac arrhythmias are frequent in patients with SARS-CoV-2 infection and AF is the most common [76, 77, 78, 84, 85, 86, 87, 88]. In a meta-analysis recently published by Romiti *et al*. [79], prevalence of AF was 8% in patients with SARS-CoV-2 infection. Risk factors associated with the arrhythmia were similar to those reported in other settings of critically ill patients, e.g., older age, male sex, common cardiovascular risk factors (hypertension, diabetes mellitus), and comorbidities such as heart failure. In line with records from other settings [22, 26], prevalence of the arrhythmia was higher in patients with worse clinical conditions. As Lip *et al*. [80] noticed in a prospective cohort study among elderly patients, the weight of classic risk factors was lower than COVID-19 infection alone in AF development. Thus, the incident AF showed the highest association with COVID-19 infection, while heart failure, coronary artery disease, and valvular disease seemed less influential.

Several mechanisms have been proposed to describe the association between COVID-19 and AF, and a considerable amount is in common with other acute diseases: inflammation, direct viral damage to cardiomyocytes, vasoactive molecules release, endothelial damage, hypoxemia, electrolytic imbalances [85, 89]. Similarly, even in the setting of COVID-19, AF is associated with a trend in increased morbidity and mortality [79, 90, 91].

It is noteworthy that COVID-19 disease is associated with a higher either arterial or venous thrombotic risk, which is important to keep in mind in the therapeutic management of these patients [92]. In this regard, in a cohort of outpatients with COVID-19 infection and cardiometabolic diseases it has been observed that the use of direct oral anticoagulants was associated with a lower rate of both arterial and venous thrombotic events as compared to vitamin K antagonists use [81, 82].

Long term data on the association between AF and SARS-CoV-2 infection are still lacking, but it is conceivable they will be available in the next years.

## 7. Conclusions

AF is common in the setting of acute and critical illnesses, including pneumonia.

Despite this well-established correlation, at present few studies explored the topical issue of AF in the setting of pneumonia. Newer data would be of great interest in order to support practical management of patients, especially in the ever-growing need of better resources allocation: knowing patients who are at major risk of developing AF could guide clinicians in choosing who could best benefit from electrocardiographic-monitoring, in preferring antibiotics that are less arrhythmogenic and in having extra care in avoiding electrolyte imbalances and AF predisposing factors. Above all, clinicians should be aware to treat AF during pneumonia not as a bystander, as previously hypothesized, but instead as AF tout court, as it is.

In the specific setting of acute pneumonia observational data indicate that new-onset AF is associated with AF recurrences, as well as a risk of stroke and mortality. Even if no randomized controlled studies have investigated this specific setting, the consensus document from EHRA recommends to follow the general approach to thromboprophylaxis for acute illness, and consider long-term treatment with oral anticoagulants in patients at risk, taking into account the anticipated net clinical benefit of anticoagulation and patient preferences.
